# Structure Model Index Does Not Measure Rods and Plates in Trabecular Bone

**DOI:** 10.3389/fendo.2015.00162

**Published:** 2015-10-13

**Authors:** Phil L. Salmon, Claes Ohlsson, Sandra J. Shefelbine, Michael Doube

**Affiliations:** ^1^Bruker micro-CT, Kontich, Belgium; ^2^Center for Bone and Arthritis Research, Institute of Medicine, University of Gothenburg, Gothenburg, Sweden; ^3^Department of Mechanical and Industrial Engineering, Northeastern University, Boston, MA, USA; ^4^Department of Bioengineering, Imperial College London, London, UK; ^5^Skeletal Biology Group, Department of Comparative Biomedical Sciences, The Royal Veterinary College, London, UK

**Keywords:** SMI, EF, geometry, ellipsoid, measurement

## Abstract

Structure model index (SMI) is widely used to measure rods and plates in trabecular bone. It exploits the change in surface curvature that occurs as a structure varies from spherical (SMI = 4), to cylindrical (SMI = 3) to planar (SMI = 0). The most important assumption underlying SMI is that the entire bone surface is convex and that the curvature differential is positive at all points on the surface. The intricate connections within the trabecular continuum suggest that a high proportion of the surface could be concave, violating the assumption of convexity and producing regions of negative differential. We implemented SMI in the BoneJ plugin and included the ability to measure the amounts of surface that increased or decreased in area after surface mesh dilation, and the ability to visualize concave and convex regions. We measured SMI and its positive (SMI^+^) and negative (SMI^−^) components, bone volume fraction (BV/TV), the fraction of the surface that is concave (CF), and mean ellipsoid factor (EF) in trabecular bone using 38 X-ray microtomography (XMT) images from a rat ovariectomy model of sex steroid rescue of bone loss, and 169 XMT images from a broad selection of 87 species’ femora (mammals, birds, and a crocodile). We simulated bone resorption by eroding an image of elephant trabeculae and recording SMI and BV/TV at each erosion step. Up to 70%, and rarely <20%, of the trabecular surface is concave (CF 0.155–0.700). SMI is unavoidably influenced by aberrations induced by SMI^−^, which is strongly correlated with BV/TV and CF. The plate-to-rod transition in bone loss is an erroneous observation resulting from the close and artifactual relationship between SMI and BV/TV. SMI cannot discern between the distinctive trabecular geometries typical of mammalian and avian bone, whereas EF clearly detects birds’ more plate-like trabeculae. EF is free from confounding relationships with BV/TV and CF. SMI results reported in the literature should be treated with suspicion. We propose that EF should be used instead of SMI for measurements of rods and plates in trabecular bone.

## Introduction

Since Hildebrand and Rüegsegger published their *structure model index* (SMI) method in 1997 (1) it has become the *de facto* standard for measuring the rod- and plate-like geometry in 3-dimensional trabecular bone images. “Rods” are regions of trabecular bone characterized by being elongated and cylindrical, whereas “plates” are extensive, flatter regions. The relative proportion of rods to plates in trabecular bone is thought to be important for bone’s mechanical competence, with plates considered to be mechanically superior to rods. SMI is straightforward to implement and is built into X-ray microtomography (XMT) analytical software, including that of commercial vendors (notably SkyScan and SCANCO) and the free BoneJ plugin for ImageJ (2). The principle of SMI is very simple: it compares the surface area of a structure before and after an infinitesimal dilation, to its volume. For primitive, convex geometries such as flat planes (SMI = 0), cylindrical rods (SMI = 3), and spheres (SMI = 4) SMI works well. Unfortunately, SMI does not account for concave surfaces, possibly making it unsuitable for use on bone geometries. For trabecular bone to form a network of two distinct and continuous phases, bone and marrow space, it must contain abundant concave surfaces joining the elements in addition to convex surfaces forming the elements themselves. Concave surfaces can exist as saddle curves (concave in one direction and convex in the other), troughs (concave in one direction and linear in the other), or bowls (concave in two orthogonal planes). To calculate SMI, a mesh of triangles is fitted to the bone surface and its area is calculated as the sum of the areas of all the triangles (*S*). The mesh is then dilated a short distance (*r*) away from the surface in the direction of the triangles’ vertex normals and surface area measured again. An approximal surface area derivative is calculated by dividing the change in surface area (*S*′) by *r*, and multiplied with bone volume (*V*) to give a final SMI value (Eq. 1).

(1) $$\text{SMI}=\text{6}\cdot \frac{\frac{S\prime }{r}\cdot V}{S^{\text{2}}}$$

Crucially, triangles on concave surfaces shrink in area when the volume is dilated, leading to a negative change in area and a negative contribution to *S*′. The negative contribution is an aberration from the mathematical and conceptual formulation of SMI, the presence of which contaminates the final SMI determination.

Because SMI is set up to report a single figure summarizing an entire bone surface, an SMI of 0 (ostensibly plate-like) can result from a surface that has an SMI of +1 on its convex portions and −1 on its concave portions. Sometimes, the SMI sum is less than its defined minimum bound of 0 (3–15), which casts serious doubt upon the biological interpretation that is possible from any SMI result. The limitations in SMI have been known since its inception: Hildebrand and Rüegsegger (1997) wrote that “*the intersections between structure elements, e.g., between rods and plates, are not accounted for*,” and their Figure 2 displays a consistent (but not discussed) negative relationship between SMI and bone volume fraction (BV/TV) (1). Despite these inherent shortcomings, SMI has been used as core experimental evidence by the bone community, having been cited over 800 times to date according to Google Scholar^1^. SMI data have been used in support of denosumab (16), bisphosphonates (3, 17–21), teriparatide (21–24), strontium ranelate (25), rosiglitazone (26), propranolol (27), and newly discovered molecules (28, 29) as treatments to improve bone architecture in human patients. It is well known that SMI is strongly confounded by BV/TV (1, 30) [*r* = −0.80 (31, 32), *r* = −0.76, −0.93 (33)*, R*^2^ = 0.86 (34), *R*^2^ = 0.94 (10), *R*^2^ = 0.80 (4), *R*^2^ = 0.91 (35)], so that comparisons of SMI values can be valid only if BV/TV is identical among specimens. This raises problems for studies where BV/TV variation is a prominent feature, for example, those of mechanical (un)loading, osteoporosis, or ovariectomy animal models. Our concern is that the pervasive use and misunderstanding of SMI has lead to many erroneous conclusions relating to plate-rod transitions in bone physiology and pathology. Until recently, there has been no widely available, validated and meaningful alternative to SMI, and so its use has continued despite its severe limitations.

Ellipsoid factor (EF) has been developed as a method for measuring rod- and plate-like geometry, independent of other geometric parameters, by fitting maximal inscribed ellipsoids inside 3-dimensional continua (36). Early experience suggests that EF effectively identifies rods and plates directly by fitting elongated (prolate) or flattened (oblate) ellipsoids, respectively, and identifies transitional regions with rounder, intermediate ellipsoids. EF at each point is calculated from the ellipsoid of greatest volume that encloses the point and that fits inside the structure. EF is calculated from the maximal ellipsoid’s semi-axes *a*, *b*, *c*:

(2) $$a\leq b\leq c,\text{ EF}=\frac{a}{b}-\frac{b}{c}$$

Bony plates are approximated by discus-shaped ellipsoids, where *a* ≪ *b* ≤ c and EF → −1; rods are approximated by javelin*-*shaped ellipsoids where *a* ≤ *b* ≪ *c* and EF → 1, whereas transitional regions have less extreme geometry and are rugby ball-shaped where *a* ≤ *b* ≤ *c* and EF → 0. A special class of ellipsoid exists for which *a*/*b* = *b*/*c*, where *a* = *qb* = *q*^2^*c*; spheres have *q* = 1.

We aim to investigate the source of the relationship between SMI and BV/TV, to determine whether SMI measures rods and plates in real trabecular bone geometries, and to quantify the amount of concave bone surface typically present in trabecular bone. Additionally, we use this opportunity to perform further testing of BoneJ’s new EF feature to determine whether it is free of significant confounding effects and could be used in place of SMI when rod/plate geometry needs to be measured.

## Materials and Methods

Two image data sets from the authors’ previous work were analyzed using SkyScan’s CTAn (v1.4; Bruker, Kontich, Belgium) and BoneJ [v1.4.1 (37); bonej.org (2)] in ImageJ [v1.49q (38)]. The data composed XMT images of trabecular bone in rat femoral metaphyses (*n* = 38) from a study investigating sex steroid rescue of ovariectomy-induced bone loss (39), and XMT images (*n* = 169) of trabecular bone in the femoral head and/or condyle from 87 species (69 mammals, 17 birds, and one crocodile), made for a study of trabecular bone scaling (40). The rat images were included to provide data from a large number of similar geometries formed under experimental conditions relevant to typical small animal studies on trabecular bone, which would test the response of SMI to generalized bone resorption and apposition. The taxonomically broad images were included to provide a diverse range of naturally occurring trabecular bone geometries, which would test the response of SMI to many different real trabeculae. Specimen data are provided as Supplementary Material. All procedures on the rats were approved by the ethics committee at the University of Gothenburg and conformed to the National Institutes of Health Guide for the Care and Use of Laboratory Animals.

Rat metaphyses were imaged at 7.66 μm isotropic pixel spacing in a SkyScan 1072 XMT scanner (Bruker, Kontich, Belgium). The interspecies trabecular series was imaged at 3.4–15 μm isotropic pixel spacing (depending on specimen size) in an X-Tek HMX ST 225 XMT scanner (Nikon Metrology, Tring, UK). Full details of image acquisition can be found in the original manuscripts (39, 40). The rat trabeculae were selected by manually drawing a conforming volume of interest distal to the growth plate that excluded cortical bone. The interspecies images were cropped down to cuboid stacks containing trabecular bone and marrow only. Binary images were produced by automatic thresholding with ImageJ’s default IsoData algorithm (interspecies) or an adaptive local thresholding algorithm (rats) (41).

The rat image series was analyzed using CTAn and again with BoneJ to generate a comparison of their SMI implementations. BV/TV was measured on the rat bones in CTAn. The interspecies series was analyzed solely with BoneJ. BoneJ has the unique feature of calculating the positive (SMI^+^) and negative (SMI^−^) components of SMI by determining the triangles that increase or decrease in area, respectively, during surface mesh dilation. The SMI equation (1) can be expanded into each triangle’s contribution (*s*′) to the overall change in surface area (*S*′)

(3) $$\text{SMI}=\text{6}\cdot \frac{V}{r}\cdot \left(\frac{s\prime_{\text{1}}}{S^{\text{2}}}+\frac{s\prime_{\text{2}}}{S^{\text{2}}}+\frac{s\prime_{\text{3}}}{S^{\text{2}}}+\ldots \frac{s\prime_{\text{n}}}{S^{\text{2}}}\right)$$

Grouping of the positive (*t*′) and negative (*u*′) values of *s*′ allows calculation of SMI^+^ and SMI^−^, respectively

(4) $$\begin{array}{l} {\text{SMI}}^{\text{+}}=\text{6}\cdot \frac{V}{r}\cdot \left(\frac{t\prime_{\text{1}}}{S^{\text{2}}}+\frac{t\prime_{\text{2}}}{S^{\text{2}}}+\frac{t\prime_{\text{3}}}{S^{\text{2}}}+\ldots \frac{t\prime_{\text{p}}}{S^{\text{2}}}\right),\;\\ {\text{SMI}}^{\text{-}}=\text{6}\cdot \frac{V}{r}\cdot \left(\frac{u\prime_{\text{1}}}{S^{\text{2}}}+\frac{u\prime_{\text{2}}}{S^{\text{2}}}+\frac{u\prime_{\text{3}}}{S^{\text{2}}}+\ldots \frac{u\prime_{\text{q}}}{S^{\text{2}}}\right) \end{array}$$

and it follows that

(5) $$\text{SMI}={\text{SMI}}^{\text{+}}+{\text{SMI}}^{\text{-}}$$

BoneJ displays in 3D the surface mesh with triangles color-coded according to the sign and magnitude of *s*′ relative to individual triangle area. The proportion of the surface area that is concave (the “concave fraction,” CF) can be calculated as the proportion of the total surface area (*S*) covered by triangles that decrease in area during mesh dilation (*U*)

(6) $$\text{CF}=\frac{U}{S}$$

Full details of BoneJ’s SMI implementation can be read in the StructureModelIndex class in BoneJ’s source code. Meshing was performed according to the standard marching cubes algorithm (42) as implemented in the ImageJ 3D Viewer (43) on binary images with a threshold of 128, voxel resampling of 3, and mesh smoothing of 0.5.

Ellipsoid factor was implemented in BoneJ with some minor performance enhancements on its initial release (36). Briefly, for each point of the structure’s medial axis, a small ellipsoid is seeded and iteratively dilated, rotated, and translated slightly until no further increase in volume is achieved. Then, the largest ellipsoid containing each point in the structure is determined, and the EF calculated for that point. Mean EF values summarizing all the foreground pixels in each image were calculated and used for comparisons.

To simulate the changes in trabecular geometry during generalized bone resorption, a single binary stack of trabecular bone from an elephant’s femoral head (chosen for having thick trabeculae and relatively high BV/TV) was repeatedly eroded by 1 pixel-width across the entire bone surface with BoneJ’s Erode 3D, and SMI, SMI^+^, SMI^−^, and BV/TV measured at each step.

Pairwise comparisons were performed in R [v3.0.2 (44)] using its lm() function for linear regression. Where curved relationships were apparent in the scatter plots, quadratic curves were fitted. Quadratic fits for SMI^−^ were forced to intercept 0, because when CF is 0, SMI^−^ must be 0. No adjustment was made for multiple comparisons because we wished to test specific pairwise hypotheses, rather than gather significant results from a table of *post hoc* comparisons, or to develop a model with multiple variables and a single outcome. We also expect a degree of multicollinearity (e.g., between SMI, BV/TV, and concave fraction) and were seeking to define it rather than to avoid or correct for it.

To determine whether SMI and EF can discriminate between birds’ and mammals’ distinctive trabecular geometries, we applied a Wilcoxon–Mann–Whitney test [R’s wilcoxon.test() method] to SMI, BV/TV, CF, and EF results, with data categorized as “Mammalian” or “Avian.”

## Results

**Figure 1:**
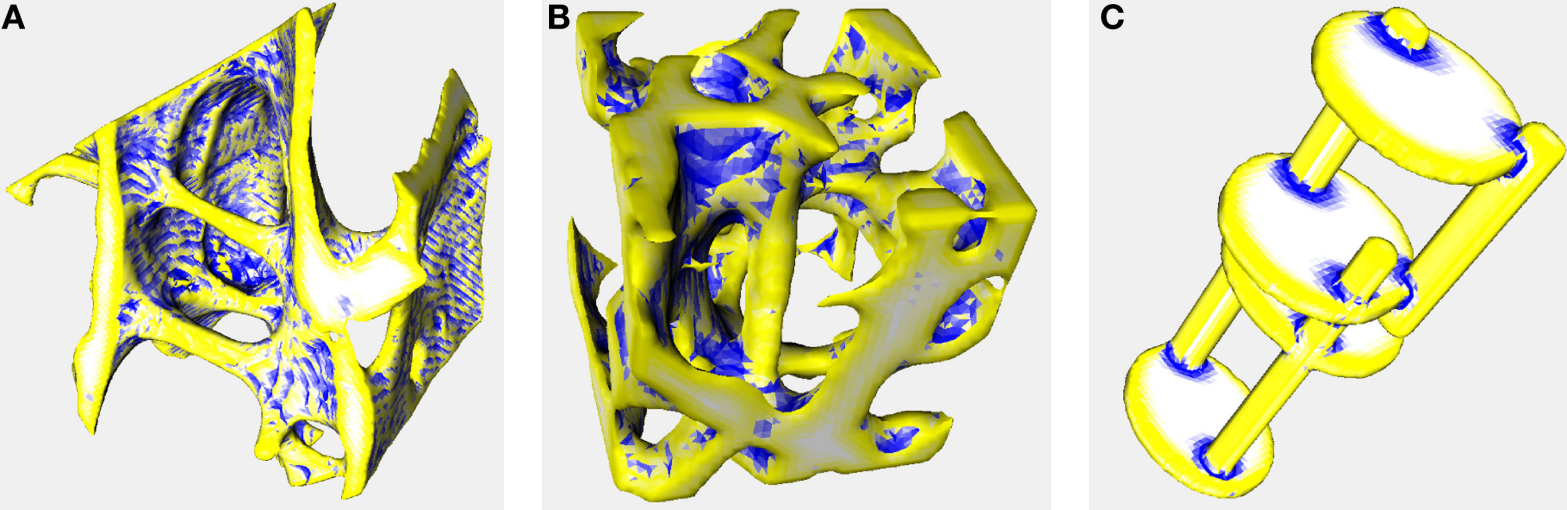
**Surface meshes color-coded by curvature**. Triangulated meshes used to calculate SMI color-coded according to whether each triangle’s area enlarged (yellow), shrunk (blue), or remained constant (white) during mesh dilation. These images were created using the same settings as the other analyses in this manuscript (voxel averaging = 3, mesh smoothing = 0.5). Yellow and white regions correspond to convex and flat curvature, respectively, that contribute to SMI^+^; blue regions correspond to concave elements that contribute to SMI^−^, artifactually decreasing SMI. Image data are the same as in Figure 2 of Doube (2015) (36). **(A)** Trabecular bone from an emu, *Dromaius novaehollandiae*. Rods, ridges, and cut edges of plates are identified in yellow indicating a positive contribution to SMI. Note the banding resulting from aliasing during mesh creation, despite pixel averaging, and mesh smoothing (CF = 0.407, SMI^+^ = 1.707, SMI^−^ = −0.617, SMI = 1.090) **(B)** Trabecular bone from a lesser dwarf shrew, *Suncus varilla*. The large vertical rod in the center of the image is correctly identified in yellow, but merges into negatively curved blue regions (CF = 0.301, SMI^+^ = 2.462, SMI^−^ = −0.493, SMI = 1.969) **(C)** Synthetic image of intersecting rods and plates. Rods and plate edges are correctly identified in yellow and plate surfaces are correctly identified in white. Junctions between rods and plates are identified in blue indicating negative contribution to SMI (CF = 0.152, SMI^+^ = 2.249, SMI^−^ = −0.315, SMI = 1.933). Full details of the implementation are available in BoneJ’s source code and input image stacks are available to download from http://bonej.org/ef.

Color-coded surface images (Figure 1) show that SMI^+^ regions (yellow) localize to rod-like features, ridges, and saddle curves with a strong convex component. SMI^−^ regions (blue) localize to depressions and junction regions.

**Figure 2:**
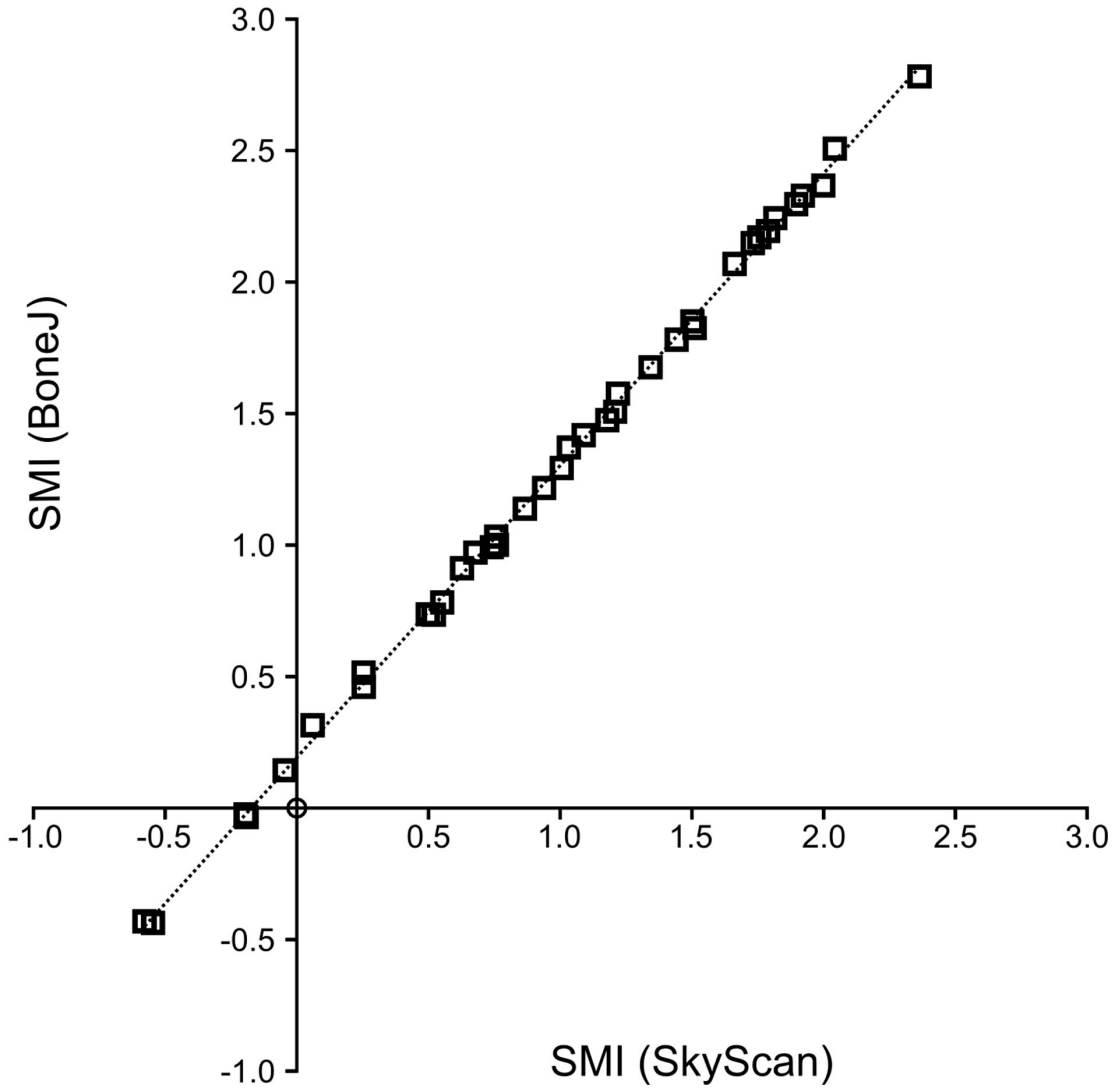
**SMI calculation by BoneJ and SkyScan**. A high level of agreement exists between BoneJ and CTAn implementations of SMI (*R*^2^ = 0.999) when run on the same image data, although BoneJ consistently overestimates SMI relative to CTAn (SMI_BoneJ_ = 1.111 × SMI_SkyScan_ + 0.192).

Structure model index values are highly correlated between CTAn and BoneJ implementations (Figure 2), so BoneJ’s results can be placed in the context of those published using commercial software with reasonable confidence.

**Figure 3:**
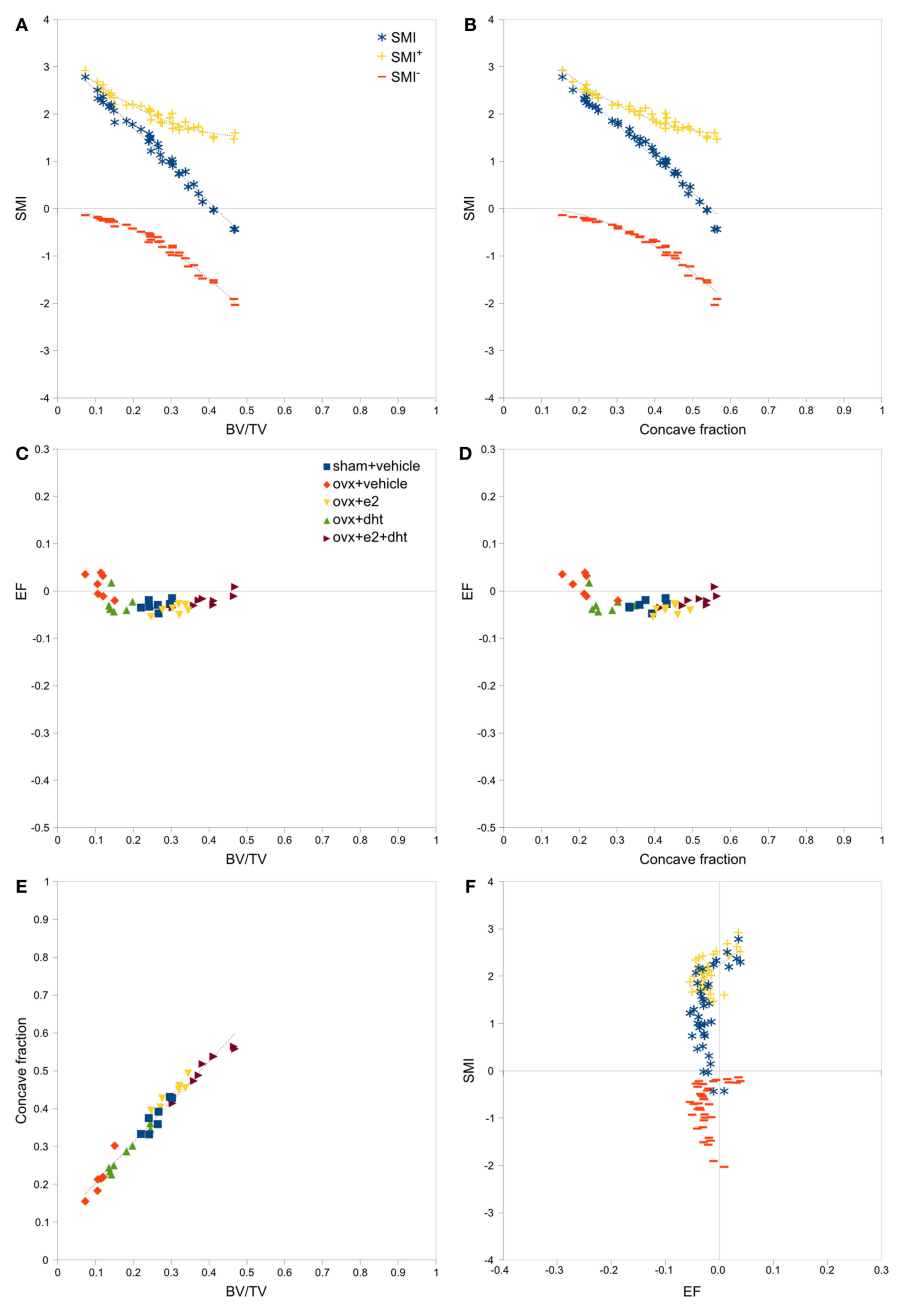
**Correlations between parameters related to SMI – rat data**. Scatterplots of pairs of parameters measured on trabeculae in rat femoral metaphyses images reveal strong and clear relationships between SMI, BV/TV and concave fraction **(A,B,E)**, while EF is independent of BV/TV **(C)**, concave fraction **(D)** and SMI **(F)**. The linear correlation between SMI and BV/TV and between SMI and concave fraction results from the sum of SMI^+^ and SMI^−^, which have complementary curved relations with BV/TV and concave fraction. Points are labeled according to experimental treatment in [**(C–E)**; ovx, ovariectomized; e2, estradiol; dht, dihydrotestosterone] and according to SMI component in **(A,B,F)**. Regression lines are plotted where *R*^2^ > 0.20, and listed in Table 1.

**Figure 4:**
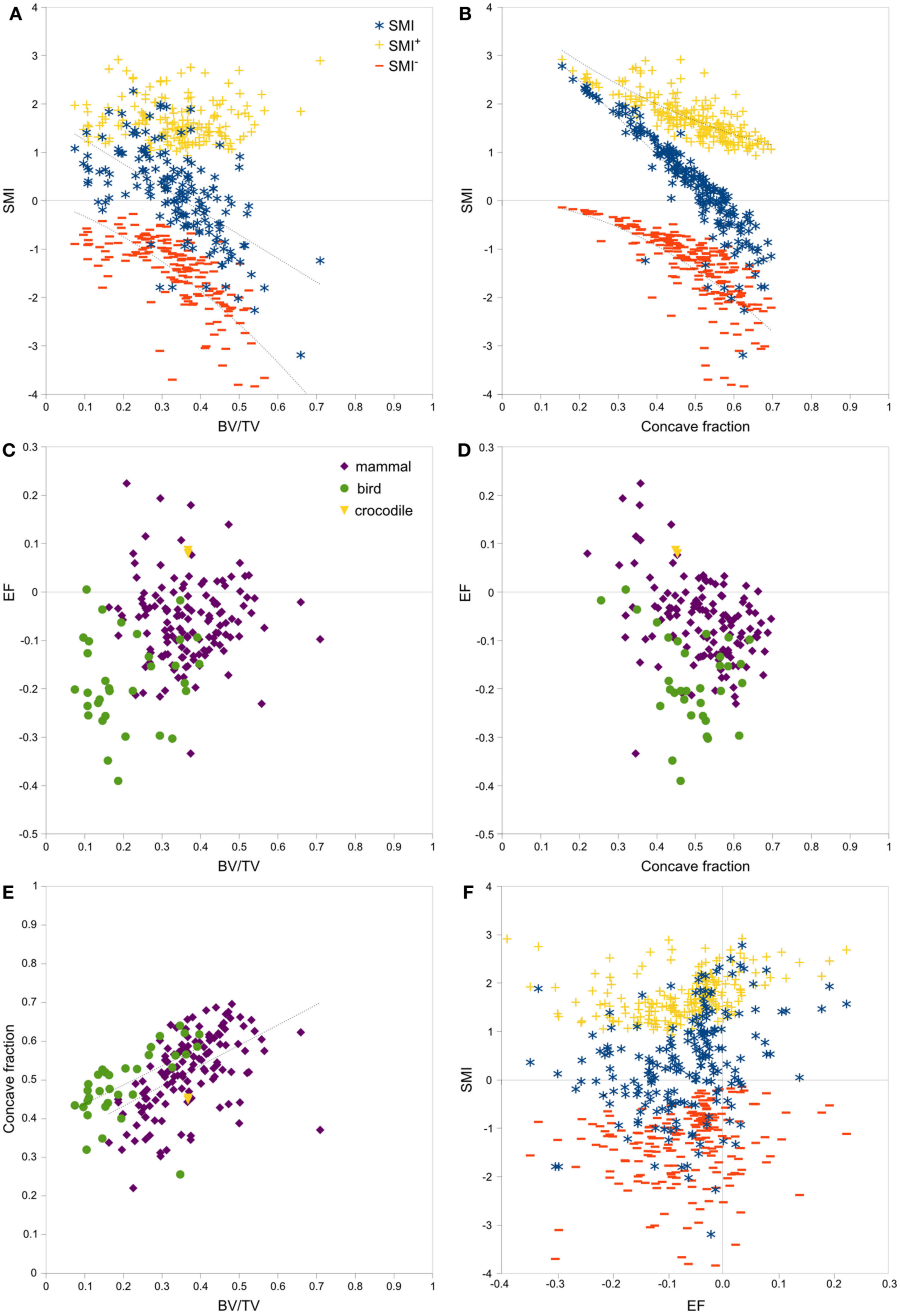
**Correlations between parameters related to SMI – interspecies data**. Scatterplots of pairs of parameters measured on trabeculae in mammal, bird and crocodile femoral head and condyle images reveal strong and clear relationships among SMI, BV/TV, and concave fraction (A,B,E). EF is nearly independent of BV/TV **(C)**, concave fraction **(D)**, and SMI **(F)** for both mammals and birds. The linear correlation between SMI and BV/TV and between SMI and concave fraction results from the sum of SMI^+^ and SMI^−^, which have complementary curved relations with BV/TV and concave fraction. Note the much broader spread of data than in the rat data in Figure 3. Points are labeled according to phylogenetic taxon in **(C–E)** and according to SMI component in **(A,B,F)**. Regression lines are plotted where *R*^2^ > 0.20, and listed in Table 1.

Strong, significant correlations implying confounded relationships, and weak correlations implying no relationship between variables, were detected in both rat (Figure 3) and interspecies (Figure 4) bone image data (Table 1). Measurements from the interspecies data were substantially more dispersed than those from the rat data, evidenced by much broader spread of points on scatter plots, lower correlation coefficients, and higher *p*-values. SMI was strongly negatively correlated with BV/TV (Figures 3A and 4A) and CF (Figures 3B and 4B), and CF was strongly positively correlated with BV/TV (Figures 3E and 4E), indicating that high BV/TV structures tend to have high CF and consequently low SMI, due to an increased contribution from SMI^−^. SMI^+^ is independent of BV/TV in the interspecies data (*R*^2^ < 0.001). EF is nearly independent of concave fraction, BV/TV, and SMI, with very low correlations for rat and interspecies data (Table 1). EF is weakly correlated with SMI^+^ in the rat data; however, the distribution of the data points (Figure 3F) and lack of relationship between EF and SMI^+^ in the interspecies data cast doubt on the ability to interpret this any further.

**Table 1:** **Regression coefficients of determination (*R*^2^) and significance levels *p* of the regressions for linear (*a* = 1) and quadratic (*a* = 2) fits to data plotted in Figures 3 and 4**.

Response variable	Explanatory variable	*a*, for *f*(*x*^a^)	*R*^2^	*p*
**Rat image data**				
SMI	BV/TV CF EF	1 1 1	0.985 0.978 0.088	<0.001 <0.001 0.039
SMI^+^	BV/TV CF EF	2 2 1	0.943 0.968 0.268	<0.001 <0.001 0.001
SMI^−^	BV/TV CF EF	2 2 1	0.995 0.989 0.003	<0.001 <0.001 0.300
EF	BV/TV CF	1 1	0.093 0.147	0.035 0.010
CF	BV/TV	1	0.974	<0.001
**Interspecies image data**				
SMI	BV/TV CF EF	1 1 1	0.249 0.555 0.096	<0.001 <0.001 <0.001
SMI^+^	BV/TV CF EF	1 2 1	<0.001 0.941 0.060	0.889 <0.001 0.001
SMI^−^	BV/TV CF EF	2 2 1	0.793 0.761 0.043	<0.001 <0.001 0.004
EF (mammal)	BV/TV CF	1 1	<0.001 0.144	0.821 <0.001
EF (bird)	BV/TV CF	1 1	<0.001 0.070	0.537 0.074
CF (mammal) CF (bird)	BV/TV BV/TV	1 1	0.262 0.288	<0.001 0.001

**Figure 5:**
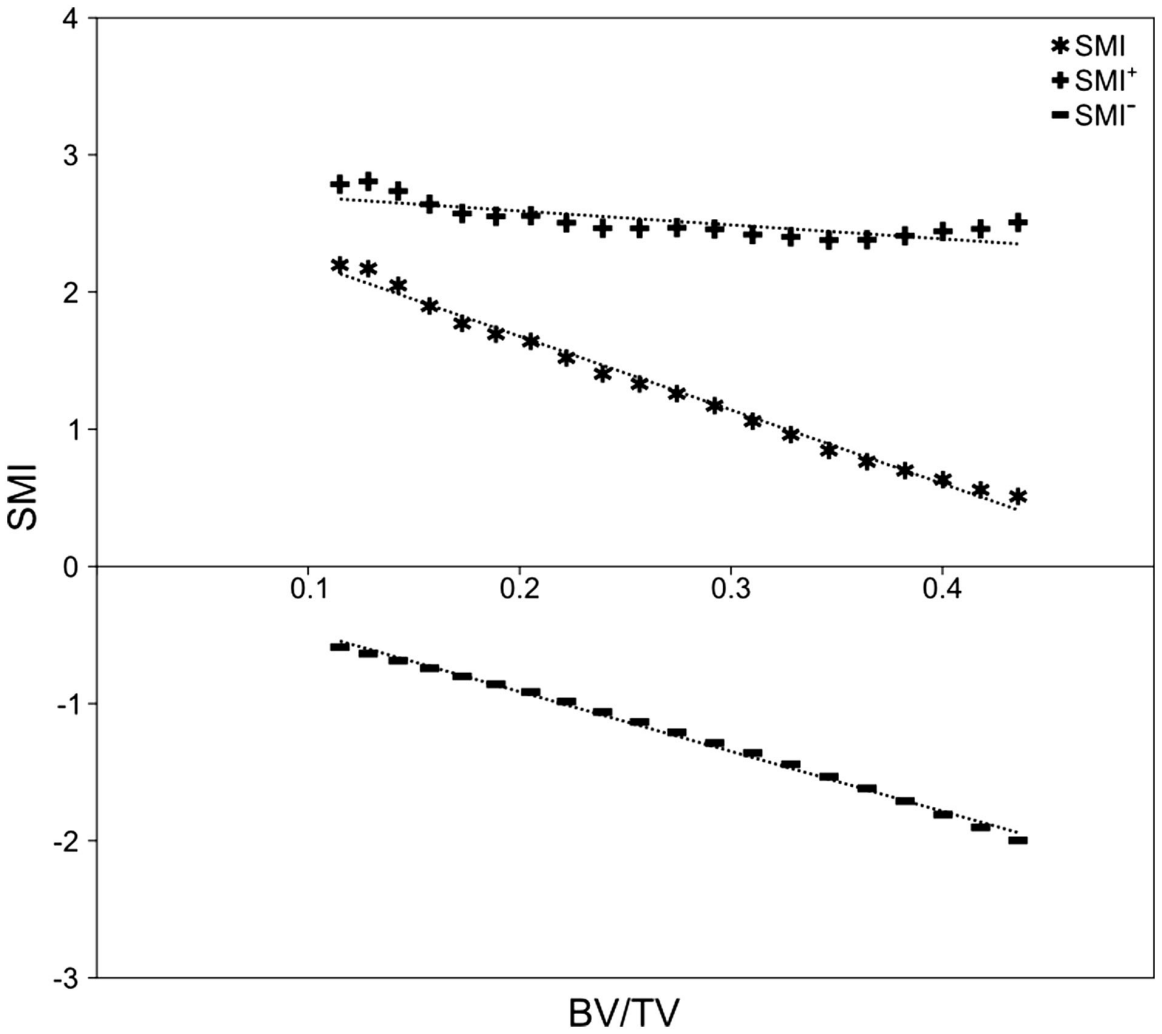
**Simulated bone resorption of elephant trabeculae**. Progressive erosion simulated bone resorption’s effect on the SMI versus BV/TV relationship. Although there is no fundamental difference in bone architecture, SMI increases from 0.51 at BV/TV of 0.44, to 2.20 at BV/TV of 0.12 (*R*^2^ = 0.991, *p* < 0.001). Note the strong negative correlation between SMI^−^ and BV/TV (*R*^2^ = 0.996, *p* < 0.001), relatively stable SMI^+^ (2.38–2.81; *R*^2^ = 0.621, *p* < 0.001), and the strong negative influence of SMI^−^ on SMI.

There was no significant difference in SMI between mammal and bird trabecular bone (*p* = 0.65), whereas EF and BV/TV were significantly different (*p* < 0.001) between mammals and birds (Figures 4C,D). Concave fraction is not significantly different between birds and mammals (*p* = 0.09) (Figures 4D,E), perhaps in part explaining the lack of discriminating ability of SMI. Sequential erosion of elephant trabeculae resulted in a nearly linear increase in SMI^−^ and SMI, and relatively stable SMI^+^, as BV/TV decreased (Figure 5).

## Discussion

Structure model index is very strongly confounded by BV/TV through the fraction of the surface that is concave, and the magnitude of the concavity. Results from our simulation and two large datasets raise the possibility that SMI does not function as intended when applied to real bone structures, and that published SMI results should not be used for assessment of plate/rod geometry. A very common interpretation of SMI results is that decreasing BV/TV is accompanied by a trend toward rod-like trabeculae, but this “trend” is merely an artifact of the design and implementation of SMI. Many studies show plots of SMI and BV/TV side by side indicating how the negative correlation can be missed and presented as though the variables are independent (9, 14, 19, 26, 27, 45–54). If a real relationship exists between decreasing BV/TV and plate-to-rod transition, SMI is too biased by concave fraction and BV/TV to measure it. A shape measure that is independent of BV/TV, such as EF, should be used instead. Because SMI does not measure rod and plate geometry in real bone structures, prior conclusions based on SMI data are at best highly suspect. We propose that past studies in which SMI results were key data are reconsidered in the light of our new findings. New studies should not use SMI.

Structure model index functions as intended only in a very small set of cases when SMI^−^ approaches 0. This occurs in artificial test images of spheres, rods and plates, such as used by BoneJ in its JUnit tests, and in bone structures with unusually low BV/TV and CF. When concave fraction approaches 0, SMI^−^ also approaches 0 and so the SMI sum nearly equals SMI^+^. The independence of SMI^+^ from BV/TV and the design of the SMI algorithm suggest that SMI^+^ could indeed measure rods and plates. Unfortunately, the original description of SMI (1) did not account for the very strong effect of SMI^−^ on the final SMI value. To be able to interpret SMI properly, it must be broken into its positive and negative components and read alongside concave fraction. This is the first study reporting the concave fraction of bone surface; however, CF has little obvious use apart from explaining why SMI is so strongly related to BV/TV. As BV/TV decreases through thinning of trabeculae, concave fraction tends to decrease in concert. SMI is strongly related to CF because as because as CF increases the contribution of SMI^−^ increases, and necessarily SMI^+^ decreases. This effect is clearer in the interspecies data than the rat data, because the interspecies data have a much weaker relation between CF and BV/TV than the rat data so their SMI versus BV/TV and SMI versus concave fraction relations are much less collinear. It also relates to geometry because it is possible to vary BV/TV without changing concave fraction; for example, a bird to mammalian transition could be modeled by packing more parallel plates of the same thickness into the same volume, increasing BV/TV while concave fraction and SMI remain the same. Readers are invited to check their image data visually for contamination by concave surfaces using the 3D option in BoneJ’s SMI implementation (Figure 1).

The fits produced on the rat data are very much tighter than those on the interspecies data, perhaps reflecting a common underlying trabecular geometry that has varied only by thickening and thinning of elements after ovariectomy and sex steroid treatment. By contrast, trabecular bone geometry varies tremendously among species. Larger mammals, cheetah, and primates tend to form extensive intratrabecular osteonal systems (40) that create large areas of tightly concave surface, which can be associated with decreased SMI (22). Birds tend toward lower BV/TV (40) and more defined plates and fine rods than mammals (Figures 1A,B). The failure of SMI to detect a significant difference between these disparate classes of animals demonstrates that it cannot be relied upon to identify even the most major differences in trabecular architecture. The interspecies data help to capture a broad parameter space within which the range of possible SMI, BV/TV, concave fraction, and EF values may lie. The rat data illustrate the relationships between variables in a scenario with direct relevance to preclinical bone research.

The very close correlation between BV/TV and SMI in our rat and simulation data could easily lead the naïve researcher to the common conclusion that decreases in BV/TV were accompanied by a dramatic shift from plate-like (SMI = 0) to rod-like (SMI > 2) trabeculae. However, it is clear when SMI is teased out into its positive and negative components, and after its relationship with concave fraction is exposed, that it is only the increasing contribution of SMI^−^ that is responsible for the apparent linkage between SMI and BV/TV, and not a plate-to-rod change in bone geometry. Mean EF does vary slightly as a function of BV/TV, but not consistently: the ovariectomized rats have a very slightly increased (more rod-like) EF, whereas all the other treatment groups have a very similar EF to the sham operated groups. The interspecies data show no relationship between EF and BV/TV and a much greater range of EF values than the rat data, reflective of the greater range of geometries present in the broad taxonomic sample. This is in clear contrast to the strong, negative, linear relationship between SMI and BV/TV, which does not relate to the real rod- or plate-like geometry of the bone structure. Increased apparent material properties associated with a rod-to-plate transition measured with SMI (10, 13, 17, 18, 26, 34, 54–57) can be explained by the close and artifactual relationship between SMI and BV/TV and the latter’s strong effect on volumetric material properties (12, 14, 33, 58, 59).

Ellipsoid factor lacks much bias or confounding relationship with BV/TV and concave fraction, but is computationally much more expensive than SMI, taking hours to days to process 1000 × 1000 × 1000 pixel images on a dual CPU, 24-core workstation (Dell T7600). Local identification of EF values allows determination of the structural source of EF summary values because individual strongly rod- or plate-like features can be visualized in the 2D stack slices or in a 3D rendering. Visualizing the spatial distribution of EF values increases confidence that the ellipsoid-fitting algorithm is correctly identifying geometries in the input image. The current implementation uses a field of 100 surface points that become relatively more sparse as the ellipsoid grows, which can mean that small features, particularly intratrabecular osteonal canals, can be ignored by the optimization algorithm. This has the effect of finding the dominant local structures but is not analytically correct. A version that uses scan-converted ellipses has shown in testing to be much more accurate but carries a significant processing time penalty in its current state and is not ready for release.

Structure model index relies upon generation of a surface mesh from 3D pixel data, which is a step that can vary by meshing algorithm implementation or by user-controlled aspects such as averaging compared to feature size. Mesh generation is a variable that is difficult to control because the smoothing and downsampling necessary to generate a smooth mesh might remove fine surface features. Even with downsampling and smoothing aliasing artifacts are visible as stripes in the surface. The relatively higher SMI values calculated by BoneJ than by CTAn may have resulted from a systematic difference in mesh creation. Mesh creation is a one-pass operation and so it should in principle scale linearly with increasing data size.

Alternative approaches to determining plate and rod architecture involve segmentation of the trabecular continuum into discrete elements, then determining the geometric qualities of each element separately (60, 61). This approach leads to binary classification of pixels into “rod” or “plate” with no recognition of transitional or junction regions. Recent work by the inventors of individual trabecula segmentation (ITS) indicates the ability of ITS-based finite element models to predict the anisotropic mechanical properties of human trabecular bone (62). An independent group found no significant contribution of ITS-based metrics to apparent material properties, which were explained almost entirely by BV/TV with a small (~10%) but significant contribution from fabric anisotropy measured by mean intercept length (MIL) (59). The ITS determination of the fraction of bone that is “plate” (pBV/BV) is strongly negatively correlated with SMI (*r* = −0.81) (61). This is somewhat concerning because it means that high pBV/BV values (i.e., plate-dominated volumes according to ITS) relate to low and ostensibly plate-like SMI values, which our current analysis leads us to believe have little meaning. This relationship might occur due to an even stronger positive correlation between pBV/BV and BV/TV (*r* = 0.99), and the already established negative correlation between SMI and BV/TV. ITS-based finite elements analysis is able to predict the off-axis mechanical behavior more closely than predictions based on BV/TV alone (62), lending support to the concept that plate and rod geometry could have a real mechanical significance. To the best of our knowledge, the ITS software is restricted by patent and not readily available as binaries or source code for others to validate. In contrast, BoneJ is open source and free for anyone to download, use, modify and redistribute.

This study exposes a broader issue of misunderstanding analysis techniques and then presenting their results as meaningful when they have failed to measure what they are generally accepted to measure. Implementation of methods by major vendors and their widespread usage creates an environment where a consensus may form, in isolation from proper reference to the details of the algorithm’s design. This style of practice creates a danger of making false conclusions with important ramifications, such as the ability of pharmaceutical or exercise interventions to improve “bone quality.”

## Conflict of Interest Statement

Dr. Phil L. Salmon is employed by Bruker micro-CT, which is a manufacturer of X-ray microtomography equipment and publisher of analytical software that was used in this work. The remaining authors have no conflict of interest to declare.

## Footnotes

^1^https://scholar.google.com/scholar?hl=en&cites=4353090427928514643

## References

[1] Hildebrand T Rüegsegger P. Quantification of bone microarchitecture with the structure model index. Comput Methods Biomech Biomed Engin (1997) 1:15–23. 10.1080/01495739708936692 11264794

[2] Doube M Kłosowski MM Arganda-Carreras I Cordelières F Dougherty RP Jackson J BoneJ: free and extensible bone image analysis in ImageJ. Bone (2010) 47:1076–9. 10.1016/j.bone.2010.08.023 20817052 PMC3193171

[3] Ito M Nishida A Aoyagi K Uetani M Hayashi K Kawase M. Effects of risedronate on trabecular microstructure and biomechanical properties in ovariectomized rat tibia. Osteoporos Int (2005) 16:1042–8. 10.1007/s00198-004-1802-3 15711780

[4] Cotter MM Simpson SW Latimer BM Hernandez CJ. Trabecular microarchitecture of hominoid thoracic vertebrae. Anat Rec (2009) 292:1098–106. 10.1002/ar.20932 19554642

[5] Skedros JG Knight AN Farnsworth RW Bloebaum RD. Do regional modifications in tissue mineral content and microscopic mineralization heterogeneity adapt trabecular bone tracts for habitual bending? Analysis in the context of trabecular architecture of deer calcanei. J Anat (2012) 220:242–55. 10.1111/j.1469-7580.2011.01470.x 22220639 PMC3381618

[6] Rubin MR Dempster DW Kohler T Stauber M Zhou H Shane E Three dimensional cancellous bone structure in hypoparathyroidism. Bone (2010) 46:190–5. 10.1016/j.bone.2009.09.020 19782782 PMC2818211

[7] Chappard D Retailleau-Gaborit N Legrand E Baslé MF Audran M. Comparison insight bone measurements by histomorphometry and μCT. J Bone Miner Res (2005) 20:1177–84. 10.1359/JBMR.050205 15940370

[8] Mócsai A Humphrey MB Ziffle JAGV Hu Y Burghardt A Spusta SC The immunomodulatory adapter proteins DAP12 and Fc receptor γ-chain (FcRγ) regulate development of functional osteoclasts through the Syk tyrosine kinase. Proc Natl Acad Sci U S A (2004) 101:6158–63. 10.1073/pnas.0401602101 15073337 PMC395939

[9] Boyd SK Davison P Müller R Gasser JA. Monitoring individual morphological changes over time in ovariectomized rats by in vivo micro-computed tomography. Bone (2006) 39:854–62. 10.1016/j.bone.2006.04.017 16757220

[10] Mittra E Rubin C Qin Y-X. Interrelationship of trabecular mechanical and microstructural properties in sheep trabecular bone. J Biomech (2005) 38:1229–37. 10.1016/j.jbiomech.2004.06.007 15863107

[11] Hunter DJ Gerstenfeld L Bishop G Davis AD Mason ZD Einhorn TA Bone marrow lesions from osteoarthritis knees are characterized by sclerotic bone that is less well mineralized. Arthritis Res Ther (2009) 11:R11. 10.1186/ar2601 19171047 PMC2688243

[12] Bayraktar HH Keaveny TM. Mechanisms of uniformity of yield strains for trabecular bone. J Biomech (2004) 37:1671–8. 10.1016/j.jbiomech.2004.02.045 15388309

[13] Teo JCM Si-Hoe KM Keh JEL Teoh SH Correlation of cancellous bone microarchitectural parameters from microCT to CT number and bone mechanical properties. Mater Sci Eng C (2007) 27:333–9. 10.1016/j.msec.2006.05.003

[14] Perilli E Baleani M Öhman C Baruffaldi F Viceconti M. Structural parameters and mechanical strength of cancellous bone in the femoral head in osteoarthritis do not depend on age. Bone (2007) 41:760–8. 10.1016/j.bone.2007.07.014 17707709

[15] Shi X Liu XS Wang X Guo XE Niebur GL. Effects of trabecular type and orientation on microdamage susceptibility in trabecular bone. Bone (2010) 46:1260–6. 10.1016/j.bone.2010.02.005 20149908 PMC2854282

[16] Reid IR Miller PD Brown JP Kendler DL Fahrleitner-Pammer A Valter I Effects of denosumab on bone histomorphometry: the FREEDOM and STAND studies. J Bone Miner Res (2010) 25:2256–65. 10.1002/jbmr.149 20533525

[17] Komatsubara S Mori S Mashiba T Ito M Li J Kaji Y Long-term treatment of incadronate disodium accumulates microdamage but improves the trabecular bone microarchitecture in dog vertebra. J Bone Miner Res (2003) 18:512–20. 10.1359/jbmr.2003.18.3.512 12619936

[18] Müller R Hannan M Smith SY Bauss F. Intermittent ibandronate preserves bone quality and bone strength in the lumbar spine after 16 months of treatment in the ovariectomized *Cynomolgus* monkey. J Bone Miner Res (2004) 19:1787–96. 10.1359/JBMR.040809 15476578

[19] Borah B Dufresne TE Chmielewski PA Johnson TD Chines A Manhart MD. Risedronate preserves bone architecture in postmenopausal women with osteoporosis as measured by three-dimensional microcomputed tomography. Bone (2004) 34:736–46. 10.1016/j.bone.2003.12.013 15050906

[20] Perilli E Le V Ma B Salmon P Reynolds K Fazzalari NL. Detecting early bone changes using in vivo micro-CT in ovariectomized, zoledronic acid-treated, and sham-operated rats. Osteoporos Int (2010) 21:1371–82. 10.1007/s00198-009-1082-z 19809776

[21] Campbell GM Bernhardt R Scharnweber D Boyd SK. The bone architecture is enhanced with combined PTH and alendronate treatment compared to monotherapy while maintaining the state of surface mineralization in the OVX rat. Bone (2011) 49:225–32. 10.1016/j.bone.2011.04.008 21515436

[22] Recker RR Bare SP Smith SY Varela A Miller MA Morris SA Cancellous and cortical bone architecture and turnover at the iliac crest of postmenopausal osteoporotic women treated with parathyroid hormone 1-84. Bone (2009) 44:113–9. 10.1016/j.bone.2008.09.019 18983947

[23] Jobke B Muche B Burghardt AJ Semler J Link TM Majumdar S. Teriparatide in bisphosphonate-resistant osteoporosis: microarchitectural changes and clinical results after 6 and 18 months. Calcif Tissue Int (2011) 89:130–9. 10.1007/s00223-011-9500-6 21626160

[24] Jiang Y Zhao JJ Mitlak BH Wang O Genant HK Eriksen EF. Recombinant human parathyroid hormone (1-34) [teriparatide] improves both cortical and cancellous bone structure. J Bone Miner Res (2003) 18:1932–41. 10.1359/jbmr.2003.18.11.1932 14606504

[25] Arlot ME Jiang Y Genant HK Zhao J Burt-Pichat B Roux J-P Histomorphometric and μCT analysis of bone biopsies from postmenopausal osteoporotic women treated with strontium ranelate. J Bone Miner Res (2008) 23:215–22. 10.1359/jbmr.071012 17922612

[26] Wei W Wang X Yang M Smith LC Dechow PC Wan Y. PGC1β Mediates PPARγ activation of osteoclastogenesis and rosiglitazone-induced bone loss. Cell Metab (2010) 11:503–16. 10.1016/j.cmet.2010.04.015 20519122 PMC3521515

[27] Khajuria DK Razdan R Mahapatra DR Bhat MR. Osteoprotective effect of propranolol in ovariectomized rats: a comparison with zoledronic acid and alfacalcidol. J Orthop Sci (2013) 18:832–42. 10.1007/s00776-013-0433-y 23835529

[28] Sashidhara KV Kumar M Khedgikar V Kushwaha P Modukuri RK Kumar A Discovery of coumarin-dihydropyridine hybrids as bone anabolic agents. J Med Chem (2013) 56:109–22. 10.1021/jm301281e 23214410

[29] Khedgikar V Kushwaha P Gautam J Verma A Changkija B Kumar A Withaferin A: a proteasomal inhibitor promotes healing after injury and exerts anabolic effect on osteoporotic bone. Cell Death Dis (2013) 4:e778. 10.1038/cddis.2013.294 23969857 PMC3763455

[30] Day JS Ding M Odgaard A Sumner DR Hvid I Weinans H. Parallel plate model for trabecular bone exhibits volume fraction-dependent bias. Bone (2000) 27:715–20. 10.1016/S8756-3282(00)00371-9 11062361

[31] Patsch JM Kiefer FW Varga P Pail P Rauner M Stupphann D Increased bone resorption and impaired bone microarchitecture in short-term and extended high-fat diet-induced obesity. Metabolism (2011) 60:243–9. 10.1016/j.metabol.2009.11.023 20171704 PMC6342255

[32] Arlot ME Burt-Pichat B Roux J-P Vashishth D Bouxsein ML Delmas PD. Microarchitecture influences microdamage accumulation in human vertebral trabecular bone. J Bone Miner Res (2008) 23:1613–8. 10.1359/jbmr.080517 18518771 PMC3276353

[33] Zhang Z-M Li Z-C Jiang L-S Jiang S-D Dai L-Y. Micro-CT and mechanical evaluation of subchondral trabecular bone structure between postmenopausal women with osteoarthritis and osteoporosis. Osteoporos Int (2010) 21:1383–90. 10.1007/s00198-009-1071-2 19771488

[34] Mittra E Rubin C Gruber B Qin Y-X. Evaluation of trabecular mechanical and microstructural properties in human calcaneal bone of advanced age using mechanical testing, μCT, and DXA. J Biomech (2008) 41:368–75. 10.1016/j.jbiomech.2007.09.003 17953972

[35] Li X Williams P Curry EJ Choi D Craig EV Warren RF Trabecular bone microarchitecture and characteristics in different regions of the glenoid. Orthopedics (2015) 38:e163–8. 10.3928/01477447-20150305-52 25760502

[36] Doube M. The ellipsoid factor for quantification of rods, plates, and intermediate forms in 3D geometries. Front Endocrinol (2015) 6:15. 10.3389/fendo.2015.00015 25762979 PMC4329874

[37] Doube M Rantalainen T Kunzelmann K-H BoneJ: BoneJ version 1.4.1. Zenodo (2015). 10.5281/zenodo.29814

[38] Schneider CA Rasband WS Eliceiri KW. NIH Image to ImageJ: 25 years of image analysis. Nat Methods (2012) 9:671–5. 10.1038/nmeth.2089 22930834 PMC5554542

[39] Tivesten Å Movérare-Skrtic S Chagin A Venken K Salmon P Vanderschueren D Additive protective effects of estrogen and androgen treatment on trabecular bone in ovariectomized rats. J Bone Miner Res (2004) 19:1833–9. 10.1359/JBMR.040819 15476584

[40] Doube M Kłosowski MM Wiktorowicz-Conroy AM Hutchinson JR Shefelbine SJ. Trabecular bone scales allometrically in mammals and birds. Proc Biol Sci (2011) 278:3067–73. 10.1098/rspb.2011.0069 21389033 PMC3158937

[41] Waarsing JH Day JS Weinans H. An improved segmentation method for in vivo microCT imaging. J Bone Miner Res (2004) 19:1640–50. 10.1359/JBMR.040705 15355559

[42] Lorensen WE Cline HE Marching cubes: a high resolution 3D surface construction algorithm. Proceedings of the 14th Annual Conference on Computer Graphics and Interactive Techniques, Anaheim, CA (1987). p. 163–9.

[43] Schmid B Schindelin J Cardona A Longair M Heisenberg M. A high-level 3D visualization API for Java and ImageJ. BMC Bioinformatics (2010) 11:274. 10.1186/1471-2105-11-274 20492697 PMC2896381

[44] Core Team R. R: A Language and Environment for Statistical Computing. Vienna: R Foundation for Statistical Computing (2013). Available from: http://www.R-project.org

[45] Roux JP Wegrzyn J Boutroy S Bouxsein ML Hans D Chapurlat R. The predictive value of trabecular bone score (TBS) on whole lumbar vertebrae mechanics: an ex vivo study. Osteoporos Int (2013) 24:2455–60. 10.1007/s00198-013-2316-7 23468074

[46] Yakacki CM Poukalova M Guldberg RE Lin A Saing M Gillogly S The effect of the trabecular microstructure on the pullout strength of suture anchors. J Biomech (2010) 43:1953–9. 10.1016/j.jbiomech.2010.03.013 20399431 PMC2900467

[47] Altman AR Tseng W-J de Bakker CMJ Huh BK Chandra A Qin L A closer look at the immediate trabecula response to combined parathyroid hormone and alendronate treatment. Bone (2014) 61:149–57. 10.1016/j.bone.2014.01.008 24468717 PMC3972893

[48] Glatt V Canalis E Stadmeyer L Bouxsein ML. Age-related changes in trabecular architecture differ in female and male C57BL/6J mice. J Bone Miner Res (2007) 22:1197–207. 10.1359/jbmr.070507 17488199

[49] Halloran BP Ferguson VL Simske SJ Burghardt A Venton LL Majumdar S. Changes in bone structure and mass with advancing age in the male C57BL/6J mouse. J Bone Miner Res (2002) 17:1044–50. 10.1359/jbmr.2002.17.6.1044 12054159

[50] Laib A Kumer JL Majumdar S Lane NE. The temporal changes of trabecular architecture in ovariectomized rats assessed by MicroCT. Osteoporos Int (2001) 12:936–41. 10.1007/s001980170022 11804020

[51] Simic P Culej JB Orlic I Grgurevic L Draca N Spaventi R Systemically administered bone morphogenetic protein-6 restores bone in aged ovariectomized rats by increasing bone formation and suppressing bone resorption. J Biol Chem (2006) 281:25509–21. 10.1074/jbc.M513276200 16798745

[52] Buie HR Moore CP Boyd SK. Postpubertal architectural developmental patterns differ between the l3 vertebra and proximal tibia in three inbred strains of mice. J Bone Miner Res (2008) 23:2048–59. 10.1359/jbmr.080808 18684086

[53] Campbell GM Ominsky MS Boyd SK. Bone quality is partially recovered after the discontinuation of RANKL administration in rats by increased bone mass on existing trabeculae: an in vivo micro-CT study. Osteoporos Int (2011) 22:931–42. 10.1007/s00198-010-1283-5 20480144

[54] Ward J Relationships of long-term bisphosphonate treatment with measures of bone microarchitecture and mechanical competence. Theses Diss – Biomed Eng (2014). Available from: http://uknowledge.uky.edu/cbme_etds/26

[55] Bevill G Eswaran SK Gupta A Papadopoulos P Keaveny TM. Influence of bone volume fraction and architecture on computed large-deformation failure mechanisms in human trabecular bone. Bone (2006) 39:1218–25. 10.1016/j.bone.2006.06.016 16904959

[56] Wegrzyn J Roux J-P Arlot ME Boutroy S Vilayphiou N Guyen O Role of trabecular microarchitecture and its heterogeneity parameters in the mechanical behavior of ex vivo human L3 vertebrae. J Bone Miner Res (2010) 25:2324–31. 10.1002/jbmr.164 20564249 PMC3179283

[57] Macrae VE Horvat S Pells SC Dale H Collinson RS Pitsillides AA Increased bone mass, altered trabecular architecture and modified growth plate organization in the growing skeleton of SOCS2 deficient mice. J Cell Physiol (2009) 218:276–84. 10.1002/jcp.21593 18803233

[58] Ashby MF. The properties of foams and lattices. Philos Transact A Math Phys Eng Sci (2006) 364:15–30. 10.1098/rsta.2005.1678 18272451

[59] Maquer G Musy SN Wandel J Gross T Zysset PK. Bone volume fraction and fabric anisotropy are better determinants of trabecular bone stiffness than other morphological variables. J Bone Miner Res (2015) 30:1000–8. 10.1002/jbmr.2437 25529534

[60] Stauber M Müller R. Volumetric spatial decomposition of trabecular bone into rods and plates – a new method for local bone morphometry. Bone (2006) 38:475–84. 10.1016/j.bone.2005.09.019 16338187

[61] Liu X Sajda P Saha P Wehrli F Bevill G Keaveny T Complete volumetric decomposition of individual trabecular plates and rods and its morphological correlations with anisotropic elastic moduli in human trabecular bone. J Bone Miner Res (2008) 23:223–35. 10.1359/JBMR.071009 17907921 PMC2665696

[62] Wang J Zhou B Liu XS Fields AJ Sanyal A Shi X Trabecular plates and rods determine elastic modulus and yield strength of human trabecular bone. Bone (2015) 72:71–80. 10.1016/j.bone.2014.11.006 25460571 PMC4282941

