# Erratum: Structure Model Index Does Not Measure Rods and Plates in Trabecular Bone

**DOI:** 10.3389/fendo.2015.00181

**Published:** 2015-11-16

**Authors:** Frontiers Production Office

**Affiliations:** ^1^Frontiers Production Office, Frontiers, Switzerland

**Keywords:** SMI, EF, geometry, ellipsoid, measurement

## Reason for Erratum

Due to a typesetting error the word “trabeculae” was changed to an incorrect spelling of “trabecule.” The publisher apologizes for this mistake and the original article has been updated. This error does not change the scientific conclusions of the article in any way.

